# Insights into Persistent SARS-CoV-2 Reservoirs in Chronic Long COVID

**DOI:** 10.3390/v17101310

**Published:** 2025-09-27

**Authors:** Swayam Prakash, Sweta Karan, Yassir Lekbach, Delia F. Tifrea, Cesar J. Figueroa, Jeffrey B. Ulmer, James F. Young, Greg Glenn, Daniel Gil, Trevor M. Jones, Robert R. Redfield, Lbachir BenMohamed

**Affiliations:** 1 Laboratory of Cellular and Molecular Immunology, Gavin Herbert Eye Institute, School of Medicine, University of California, Irvine, CA 92697, USA; fswayamp@hs.uci.edu (S.P.); skaran1@hs.uci.edu (S.K.); ylekbach@hs.uci.edu (Y.L.); 2 Department of Pathology and Laboratory Medicine, School of Medicine, University of California, Irvine, CA 92697, USA; dtifrea@hs.uci.edu (D.F.T.); figuerc1@hs.uci.edu (C.J.F.); 3 Department of Vaccines and Immunotherapies, TechImmune LLC, University Lab Partners, Irvine, CA 92660, USA; ulmerjeffrey@gmail.com (J.B.U.); drjfyoung@gmail.com (J.F.Y.); gregglenn77@gmail.com (G.G.); cdmdan@gmail.com (D.G.); trevor.m.jones@btinternet.com (T.M.J.); rrredfieldmd@gmail.com (R.R.R.); 4 Institute for Immunology, School of Medicine, University of California, Irvine, CA 92697, USA

**Keywords:** persistent, virus reservoirs, viral RNA reservoirs, long COVID

## Abstract

Long COVID (LC), also known as post-acute sequelae of COVID-19 infection (PASC), is a heterogeneous and debilitating chronic disease that currently affects 10 to 20 million people in the U.S. and over 420 million people globally. With no approved treatments, the long-term global health and economic impact of chronic LC remains high and growing. LC affects children, adolescents, and healthy adults and is characterized by over 200 diverse symptoms that persist for months to years after the acute COVID-19 infection is resolved. These symptoms target twelve major organ systems, causing dyspnea, vascular damage, cognitive impairments (“brain fog”), physical and mental fatigue, anxiety, and depression. This heterogeneity of LC symptoms, along with the lack of specific biomarkers and diagnostic tests, presents a significant challenge to the development of LC treatments. While several biological abnormalities have emerged as potential drivers of LC, a causative factor in a large subset of patients with LC, involves reservoirs of virus and/or viral RNA (vRNA) that persist months to years in multiple organs driving chronic inflammation, respiratory, muscular, cognitive, and cardiovascular damages, and provide continuous viral antigenic stimuli that overstimulate and exhaust CD4^+^ and CD8^+^ T cells. In this review, we (i) shed light on persisting virus and vRNA reservoirs detected, either directly (from biopsy, blood, stool, and autopsy samples) or indirectly through virus-specific B and T cell responses, in patients with LC and their association with the chronic symptomatology of LC; (ii) explore potential mechanisms of inflammation, immune evasion, and immune overstimulation in LC; (iii) review animal models of virus reservoirs in LC; (iv) discuss potential T cell immunotherapeutic strategies to reduce or eliminate persistent virus reservoirs, which would mitigate chronic inflammation and alleviate symptom severity in patients with LC.

## 1. Introduction

Long after the emergence of the Coronavirus disease 2019 (COVID-19) pandemic back in January 2020, many patients infected with severe acute respiratory syndrome Coronavirus 2 (SARS-CoV-2), the virus that causes COVID-19, continued to experience many and diverse lingering symptoms for months or even years following acute infection, known as Long COVID (LC), or post-acute sequelae of COVID-19 infection (PASC) [1,2,3,4,5,6,7,8,9,10,11,12,13]. A consensus definition of LC was reached in 2024 by the National Academy of Sciences as a chronic, systemic disease state with profound consequences, based on findings reported in the existing literature and patient input [2]. LC is a heterogeneous and debilitating chronic disease that currently affects at least 10 million individuals in the United States [3,4,5,6,7,8,9,10,11,12,13] and over 420 million individuals worldwide, including young infants, children, adolescents, and healthy adults [14,15,16,17,18,19,20,21,22,23,24,25,26,27,28]. With no diagnostic tests, no biomarkers, and no approved treatments currently available, the long-term global health and economic impact of chronic LC remains high and is growing [23,29,30].

**Figure 1:**
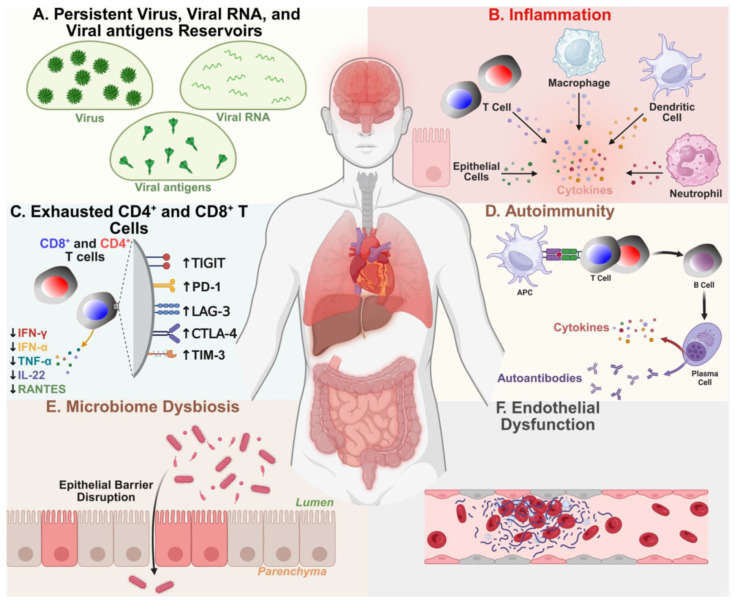
Five major mechanisms by which the virus, vRNA, and viral antigens may cause multiple and different pathologies in Long COVID patients. (**A**) A causative factor in a large subset of patients with LC is that reservoirs of virus, viral RNA (vRNA), and/or fragments may persist in multiple sites of the body. (**B**) This causes chronic inflammation, overstimulating innate and adaptive immune cells, and providing continuous viral antigenic stimuli to (**C**) exhausted CD4^+^ and CD8^+^ T cells [31,33,34,35,36]. This may result in damage to major organ systems, leading to neurological, cardiovascular, pulmonary, muscular, and psychiatric pathologies [43,44]. (**D**–**F**) Other possible causative factors of LC include metabolic disturbances, immune dysbiosis, micro-clotting, endothelial dysfunction [38,43,45,46,47], and the reactivation of HSV-1, HSV-2, EBV, CMV, and HHV-6 [48,49]. This Figure is created using BioRender.

**Figure 2:**
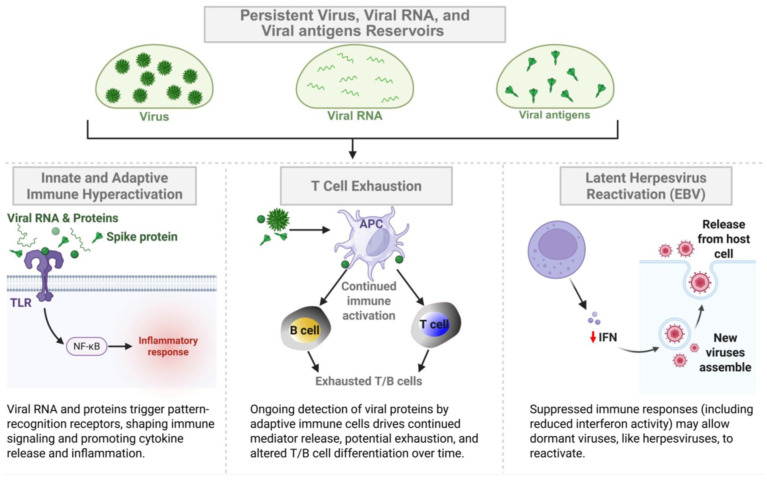
Persistent viruses, vRNA, and viral antigens may trigger the hyperactivation of both the innate and adaptive immune systems, leading to chronic inflammation, T cell exhaustion, and herpesvirus reactivation, which in turn can cause multiple and diverse pathologies in patients with Long COVID. The reservoirs of virus and/or viral RNA (vRNA) may persist and replicate in various sites of the body, driving chronic inflammation and overstimulation of immune cells [31,32,33,34,35,36,37]. Persistent reservoirs of viruses and vRNA may be capable of being translated to continuously produce viral protein antigens, either locally in affected organs or distantly released into the circulation, thereby inducing both local and systemic inflammation, immune cells overstimulation, as well as the exhaustion of CD4^+^ and CD8^+^ T cells in a subset of patients with LC [23,35,38,39,40,41,42]. Reactivation of herpesviruses, such as HSV-1, HSV-2, EBV, CMV, and HHV-6, may also be a driver of LC [48,49]. This Figure is created using BioRender.

**Figure 3:**
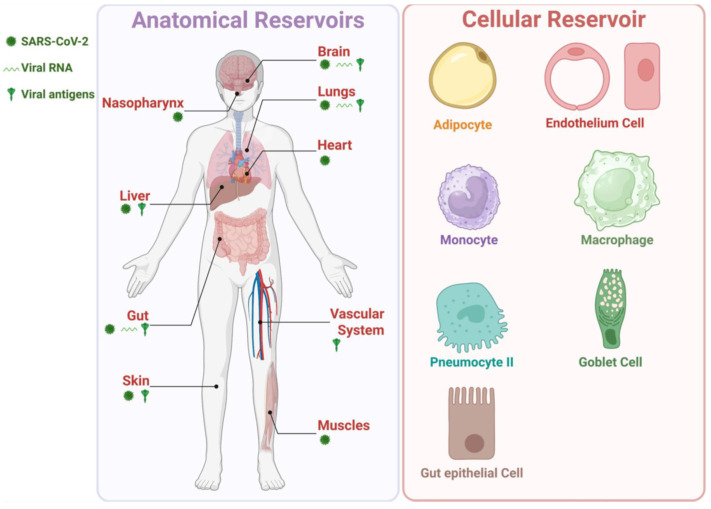
Numerous organs are affected by the virus, vRNA, and viral antigens, which persist in various cells, resulting in a range of varied pathologies in patients with Long COVID. Various anatomical locations have been identified where persistent reservoirs of virus, persistent vRNA, and, in some cases, persistent SARS-CoV-2 antigens are detected in LC patients [35,38,39,40,41,42] (**Left**). These reservoirs are detected either directly or through virus-specific immune responses that are maintained within cells from various tissues of patients with LC, long after the acute infection is cleared [23,31,33,34,35,36,50,51] (**Right**). This figure is created using BioRender.

In a proportion of patients with LC, the reservoirs of virus and/or viral RNA (vRNA) may persist and replicate in multiple sites of the body, driving chronic inflammation and overstimulation of immune cells [31,32,33,34,35,36,37] (Figure 1 and Figure 2). The virus reservoirs are characterized by the long-term persistence of pools of infected cells that harbor a replication-competent virus [31,33,34,35,36]. These persistent reservoirs of viruses and vRNA may be capable of being translated to continuously produce viral protein antigens, either locally in affected organs, or distantly released into the circulation, thereby inducing both local and systemic inflammation, immune cells overstimulation, as well as the exhaustion of CD4^+^ and CD8^+^ T cells in a subset of patients with LC [23,35,38,39,40,41,42] (Figure 2 and Figure 3).

To gain an accurate and in-depth understanding of the role of persistent virus and vRNA reservoirs in the pathophysiology of LC, this review (1) summarizes the current state of knowledge of persisting virus and vRNA reservoirs detected, either directly (from biopsy, blood, stool, and autopsy samples) or indirectly through virus-specific immune responses, in patients with LC and their association with the chronic symptomatology of LC, by reviewing a series of clinical reports from around the world, (2) explores the mechanism of inflammation and immune cells overstimulation and dysregulation that may be involved in LC, (3) discusses animal models of LC as a fundamental research tool for assessing mechanisms and targeting persistent virus reservoirs in multiple organs, (4) and deliberates potential therapeutic strategies that would reduce or eliminate persistent virus reservoirs, thereby mitigating chronic inflammation and alleviating symptom severity in patients with LC.

## 2. Long COVID Pathophysiology

**Figure 4:**
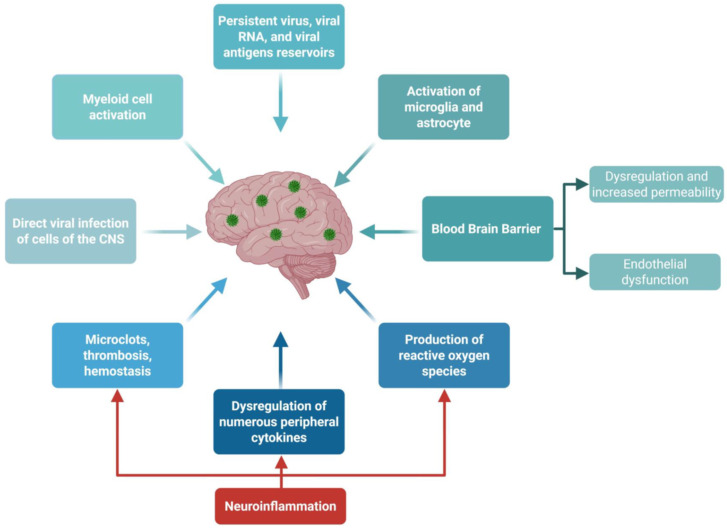
Long COVID affects the brain, nerves, and cognitive function, presenting neurological symptoms during LC. Virus reservoirs in the brain (represented as green dots) or other remote organs may activate microglia, leading to neuroinflammation and potentially contributing to cognitive symptoms in LC [92,93,94,95,96,97]. Elevated biomarkers of neurodegeneration in the cerebrospinal fluid of patients with LC suggest ongoing neuroinflammation in patients with cognitive and mental disorders, as well as psychiatric manifestations and headaches [81,100]. Persistent systemic inflammation may lead to the production of cytokines and chemokines, including IL-6, IL-8, IL-1β, TNF-α, and IP-10 [90,91], as well as the overactivation of the immune system, T cell exhaustion, and the generation of reactive oxygen species. Increased blood–brain barrier (BBB) permeability may allow cytokines to penetrate the brain and induce neuroinflammation [92,93,94,95,96,97]. A more porous BBB may also permit direct viral invasion of the brain. Tissue hypoxia may occur due to microclot formation. Neurological symptoms of LC include cognition, psychiatric manifestations, headache, and others. These conditions are more commonly described in young adults and women. This figure is created using BioRender.

Long COVID, also known as post-acute sequelae of SARS-CoV-2 infection (PASC), is a heterogeneous chronic disease that manifests three months after acute SARS-CoV-2 infection and persists for months to years [36,52,53]. Over 10% of SARS-CoV-2-infected individuals, including young infants, children, adolescents [14,26,27,28,54,55], and healthy adults, can develop LC [15,16,17,18,19,20,21,22,26,27,56,57,58,59,60,61,62,63,64,65,66,67,68,69,70,71,72,73,74,75]. LC is currently characterized by up to 200 documented symptoms that can affect 12 major organ systems, and may, in some cases, be disabling [76]. Pathologies associated with LC include neurological, cardiovascular, pulmonary, muscular, and psychiatric disorders [77,78,79,80,81,82,83,84,85]. Comorbidities encompass over 600 diseases that have been identified as increasing the risk for LC [86]. While these comorbidities span nearly all clinical specialties, they are strongly enriched in cognitive, cardiorespiratory, and endocrine-renal diseases [86]. Virus reservoirs in the brain or other remote organs may cause neuroinflammation and neurologic symptoms in patients with LC, including cognitive and mental disorders, as well as psychiatric manifestations and headaches [81] (Figure 4). Unexpected increases in antibody responses directed against non-SARS-CoV-2 viral pathogens, particularly Epstein–Barr virus, have been reported in patients with LC [87]. The underlying pathophysiological mechanisms of sex differences in the frequencies, patterns of organ system involvement, and manifestations of LC, with females being significantly more likely to develop severe LC symptoms than males [88], remain to be determined. Diverse and specific patterns of host response factors that drive the transition from acute disease to long-term chronic LC in males and females are thought to be involved in immunopathological mechanisms of LC [88,89]. Persistent systemic inflammation may lead to the production of cytokines and chemokines, including IL-6, IL-8, IL-1β, TNF-α, and IP-10 [90,91], as well as the overactivation of the immune system, T cell exhaustion, and the generation of reactive oxygen species. Increased blood–brain barrier (BBB) permeability may allow cytokines and chemokines to penetrate the brain, inducing neuroinflammation [92,93,94,95,96,97]. A more porous BBB may also permit direct viral invasion of the brain [92,93,94,95,96,97]. While many studies have identified characteristic symptom patterns of LC in adults and children older than 5 years, LC remains poorly characterized in children aged 0 to 5 years [28]. A recent multisite longitudinal cohort study identified differences in symptom patterns by age group (infants/toddlers [0–2 years] vs. preschool-aged children [3–5 years]) [26,27,98]. The study found that the symptoms of LC experienced by young children differed not only from those of adults and older children but also between age groups within early childhood, suggesting the need to characterize LC separately across all age ranges [26,27,98,99]. These symptoms differ from those experienced during Multisystem Inflammatory Syndrome in Children (MIS-C).

While the underlying causative mechanisms of LC remain to be defined, an accepted causative factor in a large subset of patients with LC, is that reservoirs of virus, viral RNA (vRNA), and/or fragments may persist and replicate in multiple sites of the body driving chronic inflammation, overstimulate innate and adaptive immune cells, and provide continuous viral antigenic stimuli to exhausted CD4^+^ and CD8^+^ T cells [31,33,34,35,36]. This may result in damage to 12 major organ systems, leading to neurological, cardiovascular, pulmonary, muscular, and psychiatric pathologies [43,44]. However, other hypotheses regarding the causative factors of LC include metabolic disturbances, immune dysbiosis, microclotting, endothelial dysfunction [38,43,45,46,47], and the reactivation of non-SARS-CoV-2 viruses, such as HSV-1, HSV-2, EBV, CMV, and HHV-6 [48,49] (Figure 1).

Below, we will review the growing body of clinical evidence that persistent reservoirs of virus, persistent vRNA, and, in some cases, persistent SARS-CoV-2 antigens in multiple organs of patients with LC, which may cause chronic inflammation and dysfunction (exhaustion) of antiviral CD4^+^ and CD8^+^ T cells associated with various symptomatology of LC [35,38,39,40,41,42].

## 3. Persistent SARS-CoV-2 Virus Reservoirs in Patients with LC

A growing number of clinical reports suggest that SARS-CoV-2 viral reservoirs persist in multiple organs of patients with LC and remain active for long periods following acute infection, contributing to the long-term chronic symptoms of LC [23,101,102]. These virus reservoirs, detected either directly or through virus-specific immune responses, are maintained by the long-term persistence of a pool of infected cells that harbor reservoirs of replication-competent virus [31,33,34,35,36] (Figure 1, Figure 2 and Figure 3). We will detail several reports that describe persisting virus reservoirs and vRNA at biopsy in patients with LC or at autopsy [101], and discuss their possible association with the symptomatology of LC.

Multiplexed imaging of post-mortem lung tissues from 12 individuals revealed evidence of viral persistence in the lungs of patients with LC, even in those with negative nasopharyngeal swabs, up to 359 days after the acute phase of the disease [103,104]. Persistent virus was detected in the appendix, skin, and breast tissues of two patients with LC, 163 and 426 days after the acute symptoms resolved [105]. A patient with LC and rheumatoid arthritis exhibited viral persistence in the nasopharynx for 6 months after the acute COVID-19 infection resolved [106]. Persistent virus reservoirs were also detected using RT-PCR, immunohistochemistry (IHC), and In Situ Hybridization (ISH) in the gastrointestinal tract (colon, gut mucosa, gut epithelium) of patients with LC, who did not clear SARS-CoV-2 after the resolution of acute infection [23,31,44,107,108]. It was proposed that long-term dysregulation of the gut in response to viral persistence may lead to myriad symptoms observed in LC [44,107,108]. Olfactory mucosa sampling at 110 to 196 days post-acute infection from long-term anosmia patients with LC, with prolonged olfactory function loss, revealed the presence of SARS–CoV–2–infected cells, along with protracted inflammation [50]. Months after acute COVID-19 resolution, nasal cytobrushes, nasal washes, and tonsillar tissue fragments were obtained from 48 children with LC undergoing testing using RT-qPCR, immunohistochemistry (IHC), and flow cytometry [109]. The study detected the presence of SARS-CoV-2 in at least one specimen of 27% of children with LC [109]. IHC for the SARS-CoV-2 non-structural protein NSP-16 indicated the presence of viral replication in 53.8% of the SARS-CoV-2-infected tissues [109]. Thus, tonsils and adenoids appeared to be major sites of persistent and replicating virus reservoir in children [109]. Several compartments of the oral cavity have been proposed as potential sites of a persistent reservoir for SARS-CoV-2 [110,111]. Evidence of SARS-CoV-2 reservoirs, accompanied by a constant stimulation of immune responses, was reported in the fungiform papillae of tongue tissue from patients with LC, 6–63 weeks after the resolution of acute COVID-19. The finding indicates a temporal association in patients between functional taste, taste papillae morphology, and the presence of SARS-CoV-2 and its associated immunological changes [112]. Few studies have detected persistent virus and vRNA in the testes and sperm of LC patients months after initial infection [113,114].

The duration of viral shedding and the maximum viral load during the acute phase correlate with the severity of subsequent LC, suggesting that individuals with a higher early viral burden may have a greater viral inoculum that persists at primary infection sites, such as the lungs or gut, or that seeds distant tissue sites [44,104,107,108,115,116,117]. The severity of the initial acute SARS-CoV-2 infection in unvaccinated, over-65, or immunocompromised patients, as well as the increased viral load during the acute phase of COVID-19 infection due to a lack of immunity, may facilitate the establishment of persistent viral reservoirs later once the acute infection clears [117]. Nearly half of the fully vaccinated patients who are hospitalized for COVID-19 symptoms were over 65 years old or immunocompromised, suggesting a role for the immune system in clearing early infections [118]. Thus, SARS-CoV-2 viral reservoirs in multiple organs of unvaccinated, over-65, or immunocompromised patients with LC may have driven chronic inflammation, immune cell overstimulation, and elevated virus-specific, exhausted T cells, which are associated with symptoms of LC [102]. Persisting virus and vRNA reservoirs are detected, either directly (from biopsy, blood, stool, or autopsy samples) or indirectly through persistent virus-specific immune responses, in patients with LC who exhibit persistent systemic inflammation months to years after the acute COVID-19 episode [119]. The dynamics of antiviral immune responses during acute infection appeared to play a role in the subsequent pathogenesis of LC, highlighting the importance of understanding early immunological markers in the natural history of LC [115]. However, the underlying mechanisms by which the virus reservoirs persist in multiple organs and lead to various symptoms of LC remain to be fully elucidated. Because the percentage of LC patients with persistent virus reservoirs, as well as the exact location and duration of these virus reservoirs in patients with LC, remains to be determined, one should not generalize persistent virus reservoirs as a cause of symptoms in all patients with LC [117].

Whether persistent virus reservoirs in patients with LC are merely an association or a cause-and-effect relationship remains to be determined in large cohorts of patients with LC and control groups and confirmed in reliable animal models of persistent virus reservoirs and LC-like symptoms, as observed in humans [120]. Whether host CD4^+^ and CD8^+^ T cells, B cells, antibodies, and innate immune cells affect the size, clonality, cellular, tissue, and organ distribution of the virus and vRNA reservoirs remains to be determined. Moreover, techniques like RNAscope, used to detect virus reservoirs in tissues of patients with LC, have sensitivity limits, especially when viral load is low or unevenly distributed, and can lead to an underestimation of viral presence. Detecting viral RNA or proteins does not necessarily indicate active virus replication. Many studies find viral fragments or antigens persisting without evidence of an infectious, replicating virus, raising questions about the nature of the reservoir and its contribution to LC symptoms. Nucleic acid-based methods can also be prone to contamination, which can affect the reliability of the results. Antigen detection methods are sometimes less sensitive or specific, requiring careful assay validation and cross-laboratory standardization. These challenges suggest that, although persistent virus and vRNA reservoirs have been identified in LC patients, more sensitive and specific methods are necessary to understand the role of these persistent virus and vRNA reservoirs in the symptomatology of LC.

## 4. Persistent Reservoirs of Viral RNA (vRNA) in Patients with LC

A growing body of clinical reports has also demonstrated the persistence of SARS-CoV-2 vRNA within cells from various tissues of patients with LC, long after the acute infection has cleared [23,51] (Figure 1, Figure 2 and Figure 3).

A persistent reservoir of SARS-CoV-2 vRNA was detected in the colon, appendix, ileum, hemorrhoids, liver, gallbladder, and lymph nodes from five patients who recovered from COVID-19, up to 180 days after testing negative for SARS-CoV-2 using vRNA in situ hybridization (RNAscope) [121]. The presence of vRNA was detected at autopsy in lung tissues from 44 patients with LC several months after the acute infection had resolved, suggesting that some LC may maintain a vRNA reservoir in the lungs through yet-to-be-determined mechanisms [104,122]. Using RNAscope, persistent vRNA was colocalized in the appendix, skin, and breast tissues of two patients with LC, 163 and 426 days after the initial infection [105]. An extensive distribution of persistent vRNA was detected at autopsy throughout the brain, as late as 230 days following symptom onset in one case [101]. Persistent vRNA reservoirs were detected in the brains of patients with LC at autopsy, up to 7 months following symptom onset [101]. The dynamics of fecal vRNA shedding were analyzed in 113 patients over 10 months, and shedding was correlated with mild-to-moderate acute LC symptoms [123]. Although there was no ongoing oropharyngeal vRNA shedding detected at 4 months post-acute infection in these patients with LC, 12.7% [8.5–18.4%] of patients with LC continued to shed vRNA in the feces at 4 months, and 3.8% [2.0–7.3%] shed at 7 months [123]. The severity of gastrointestinal symptoms (abdominal pain, nausea, and vomiting) correlated with fecal shedding of vRNA [123]. This study suggests that SARS-CoV-2 infects the gastrointestinal tract, and persistent vRNA reservoirs persist in the gastrointestinal tract of patients with LC, who did not fully clear SARS-CoV-2 after acute infection [123]. PCR analyses of intestinal biopsies obtained from patients with LC 4 months after the onset of COVID-19 revealed the persistence of vRNA in the small bowel of 7 out of 14 individuals, consistent with antigen persistence [124]. In another study, reservoirs of vRNA were detected in the gut mucosa ∼7 months after mild acute COVID-19 in 32 of 46 patients with inflammatory bowel disease (IBD) [44,107,108]. Persistent vRNA reservoirs were confirmed by another study in the stool and spinal fluid of two patients with LC, months after the acute infection had resolved [105]. Persistent vRNA in the stool of children with Multisystem Inflammatory Syndrome in Children (MIS-C) was detected up to 62 days after resolution of acute COVID-19 [125]. Long-term anosmia patients with prolonged olfactory dysfunction, as seen in LC, exhibited the presence of viral transcripts in the olfactory mucosa, accompanied by protracted inflammation [50]. Using samples from 110 children undergoing tonsillectomy and adenoidectomy, another study provides evidence of persistent tissue-specific immunity to vRNA reservoirs in adenoid and tonsil tissues for up to 303 days after the resolution of COVID-19 [126]. This study confirms previous findings that tonsils and adenoids are major sites of persistent and replicating virus reservoir in children [109].

The mechanisms by which the virus reservoir or vRNA reservoir is maintained and may contribute to various LC pathologies remain to be fully elucidated [31]. Multimodal molecular imaging in a cohort of 24 participants, spanning time points from 27 to 910 days following acute SARS-CoV-2 infection, reveals tissue-based T cell activation and vRNA persistence in patients with LC for up to 2 years following COVID-19 [102]. T-cell hyperactivation detected in the spinal cord and gut was associated with the presence of LC symptoms [44,107,108]. This suggests that persistent vRNA, which produces viral antigens for up to 2 years after acute infection is resolved, may constantly stimulate T cells, causing them to become exhausted and thereby inhibiting their ability to clear the virus reservoir at these sites.

Since the percentage of LC patients with persistent vRNA reservoirs, as well as the exact location and duration of these vRNA reservoirs in patients with LC, remains to be determined, one should not generalize persistent vRNA reservoirs as the sole cause of symptoms in all patients with LC [117]. While vRNA may reflect fragments of the SARS-CoV-2 genome that persist but are not replication-competent, growing evidence suggests that vRNA may actually represent the entire SARS-CoV-2 genome, capable of replication and producing consistent antigenic stimulation [23,35,38,39,40,41,42]. Thus, it is possible that persistent virus and vRNA reservoirs, which express viral antigens, as well as the residual viral antigens in multiple organs and circulation (e.g., Spike protein and Nucleoprotein), are behind the chronic inflammation, as well as T cell dysfunction/exhaustion, reported in many clinical studies of LC patients [23,35].

## 5. Residual SARS-CoV-2 Antigens in Patients with LC

Several reports have shown persistence of the Spike protein or its fragments within cells from various body tissues of patients with LC [23,51]. It was suggested that the Spike protein may have persisted from the initial infection. Persistent virus reservoirs and vRNA in the tissues of patients with LC can be expressed to produce viral proteins in the tissue or in circulation, inducing local or systemic chronic inflammation and causing immune cell overstimulation and T cell exhaustion [23,35,38,39,40,41,42]. Residual viral protein antigens (i.e., Spike protein, Nucleoprotein, and other viral antigens) persist within cells in various organs (gut, brain, tonsils, lungs, heart, or reproductive organs) and the circulation months after the acute COVID-19 infection is resolved [23,35,38,39,40,41,42,44,51,107,108,127,128].

### 5.1. Residual Spike Protein Is Associated with LC Symptoms

As of September 2025, worldwide, there have been over 789 million confirmed COVID-19 cases [15,16,17,18,19,20,21,22,117,118,129,130,131,132,133,134,135,136,137,138,139,140,141,142,143,144,145,146,147,148,149,150,151,152,153]. Patients who develop LC have persistent Spike protein present, exacerbated by multiple exposures to SARS-CoV-2 infections over the last 5 years.

An endoscopy study performed in 46 LC patients with inflammatory bowel disease (IBD) revealed persistent viral antigens in the gut 219 days after a confirmed COVID-19 infection, and these were associated with severe LC symptoms [44,107]. Persistent Spike protein and S1 subunit were detected in unvaccinated patients with LC compared with vaccination-matched non-LC controls [87]. Similarly, Peluso et al. detected SARS-CoV-2-specific T cell activation in the gut up to 2.5 years after acute SARS-CoV-2 infection, suggesting persistence of viral antigen in tissues [102]. Another study has shown that persistent SARS-CoV-2 peptide fragments in multiple organs may drive inflammation by mimicking the action of specific immune molecules in the body [154]. Optical clearing and imaging revealed localized accumulation of Spike protein in the skull–meninges–brain axis of human COVID-19 patients, persisting 12 months after acute viral clearance [100]. This was associated with elevated biomarkers of neurodegeneration in the cerebrospinal fluid of patients with LC, suggesting ongoing neuroinflammation in these patients. Proteomic analysis of human skull, meninges, and brain samples revealed dysregulated inflammatory pathways and neurodegeneration-associated changes [100]. These findings suggest that persistent Spike protein in the brain may contribute to lasting neurological sequelae of LC [100]. Similarly, in another study, both Spike protein and vRNA were found in 30% of patients with LC, while none of the control individuals without LC were found to have these [155]. Case reports indicate that the Spike protein and vRNA appeared to persist in bronchoalveolar lavage from patients with LC for up to two years after acute SARS-CoV-2 infection [104,105]. Using multiplex immunohistochemistry, SARS-CoV-2 nucleoprotein was detected in the appendix, skin, and breast tissues of two patients with LC, 163 and 426 days after the onset of acute LC symptoms [105]. A persistent Spike protein and S1 subunit within CD16+ monocytes have been proposed as potential drivers of the pathophysiology of LC [156,157,158,159] (Figure 1, Figure 2 and Figure 3).

Other case reports indicate that the Spike protein can be detected in the tissues of patients with LC up to a year after infection [93,97,105,129,130,131,132,133,134,135,136,137,138,139,140,141,142,143,144,145,146,147,148,149,150,151,152,153,157,158,160,161,162,163,164,165]. One study reported that the presence of recombinant Spike protein has been detected in the blood of individuals who received the mRNA Spike protein-based vaccine up to 3 months post-vaccination, regardless of antibody titer [166]. The study employed mass spectrometry analysis of biological samples to detect the presence of specific fragments of the recombinant Spike protein in subjects who received mRNA-based vaccines [166]. The minimum and maximum times at which the Spike protein was detected after vaccination were 69 and 187 days, respectively. Other studies have also reported the persistence of the Spike protein in patients with LC for 6 [166] and 15 months post-acute COVID-19 infection, with the absence of viable virus confirmed by negative PCR and RNA assays [156]. Spike protein and nucleoprotein were both detected in the colon, appendix, ileum, hemorrhoids, liver, gallbladder, and lymph nodes from five patients who recovered from COVID-19, up to 180 days after testing negative for SARS-CoV-2 using conventional immunohistochemistry [121]. However, the study was unable to detect vRNA in some patients’ tissues, possibly due to a higher mRNA degradation rate compared to protein, and the timing of detection, which occurred after recovery from acute infection [121]. Circulating Spike protein linked to extracellular vesicles with and without vRNA fragments appeared to persist in patients with LC up to one year after acute SARS-CoV-2 infection [128,155]. Finally, a study detected the Spike protein 219 days after the original positive endoscopy in the gut lining of 15 out of 132 subjects, despite the absence of replicating SARS-CoV-2 cultured from these patients’ gut tissues, which showed viral antigen persistence [107]. Residual Spike, the protein S1 subunit, was detected in patients with LC 8 and 12 months after COVID-19 resolved [128,167]. These data suggest that the circulating Spike protein and its S1 subunit may serve as a potential biomarker for persistent viral reservoirs [128,167].

Many of the above studies detecting virus reservoirs, vRNA reservoirs, and residual antigens in patients with LC are limited by small patient cohorts [168]. A recent study compared residual antigens at multiple time points following acute infection in a larger cohort of pandemic-era LC patients with control adults collected before 2020 (pre-pandemic era) [168]. By using the Simoa (Quanterix) single-molecule array detection platform, the study measured residual Spike protein, S1 subunit, and nucleoprotein antigens. It showed 61 (9.2%) specimens from 42 participants (25%) contained one or more detectable SARS-CoV-2 antigens [117,168]. The most commonly detected antigen was Spike protein (*n* = 33, 5.0%), followed by S1 subunit (*n* = 15, 2.3%) and nucleoprotein [117,168]. The study provides strong evidence that virus reservoirs, vRNA reservoirs, and/or residual antigens may persist in some form or location of patients with LC for up to 14 months following the resolution of acute SARS-CoV-2 infection [117,168]. However, the study cautions that the findings provide no direct evidence regarding the persistent presence of replication-competent or transcriptionally active virus, nor that it causes LC [117,168].

As of September 2025, more than 15.9 billion COVID-19 vaccine doses (mostly Spike-based mRNA vaccines) have been administered worldwide [135,147,169]. COVID-19 vaccines not only prevent acute COVID-19 morbidity and mortality but also significantly reduce the risk of developing persistent LC symptoms [170,171,172,173,174]. Compared to individuals who received complete COVID-19 vaccination, unvaccinated individuals showed an increase in virus load and COVID-19 morbidity, which may have led subsequently to a significant increase in the incidence of LC [175]. The protective effect of the COVID-19 vaccines appears to be particularly robust when vaccination occurs before infection, though benefits have also been observed in preventing LC symptom progression in breakthrough cases [176,177,178,179,180,181,182].

Few small studies on the biodistribution patterns of the Spike protein following mRNA vaccines raised questions about whether persistent Spike in organs outside the site of administration could be responsible for some of the LC symptoms in immunocompetent patients [93,128,183,184,185,186,187]. While extremely rare in humans, in animal models of stroke and traumatic brain injury, the administration of Spike protein alone was sufficient to induce neuroinflammation, proteome changes in the skull–meninges–brain axis, anxiety-like behavior, and exacerbated outcomes [100]. Vaccination reduced but did not eliminate Spike protein accumulation after infection in mice [100]. Reports also indicate that the Spike protein may damage the endothelium in animal models, disrupt an in vitro model of the blood–brain barrier (BBB), and cross the BBB, leading to perivascular inflammation [92,93,94,95,96,97]. It was hypothesized that the Spike protein entering the brain or being expressed by brain cells could activate microglia, leading to neuroinflammation and potentially contributing to cognitive symptoms in LC [92,93,94,95,96,97]. These findings suggest persistent Spike protein at the brain borders post-vaccination may contribute to lasting neurological sequelae of LC [100]. Randomized placebo-controlled clinical trials are currently underway to confirm or refute the observations and hypotheses regarding Spike persistence at months 1, 3, 6, and 12 in vaccine and control arms, as well as to assess the benefits of COVID-19 vaccination in reducing LC symptoms.

One study showed that out of 200 unvaccinated COVID-19 convalescent individuals, 21.5% (*n* = 43) presented cardiac, pulmonary, muscular, and psychiatric symptoms three months post-infection, had decreased S1 subunit, S2 subunit, and nucleoprotein-specific IgG antibodies [43,188]. Other studies showed that patients with LC have circulating Spike protein and Spike protein-specific antibodies one year after infection or vaccination [128,188,189,190,191]. Similarly to the potential involvement of the Spike protein in LC, it was hypothesized that the persistence of the S1 subunit in CD16^+^ monocytes up to 245 days post-acute infection sustains chronic inflammation, which may contribute to the duration of symptoms in some vaccinated patients with LC [159]. Computational sequence analysis of the Spike protein revealed (i) a super antigen (SAg)-like motif highly similar to a Staphylococcal enterotoxin B (SEB) fragment in the Spike protein subunit S1 with in silico high affinity for binding T cell receptors (TCRs) and MHC Class II [192,193]. This prompted a hypothesis of autoimmunity leading to the development of LC [192,193], and (ii) conserved snake neurotoxin-like motifs, which may alter neuronal cell function and contribute to neurological symptoms [192,193].

A report indicates that the contribution of the Spike protein S1 subunit to lung inflammation is mediated by the NLRP3 inflammasome machinery and the release of cytokines, including interleukin-6 (IL-6), IL-1β, and IL-18 [104,194]. Matrix Metalloproteinase-9 (MMP-9) was significantly elevated in the serum of patients with LC compared to healthy controls. The Spike protein appeared to stimulate microglia in vitro to produce MMP-9, which may contribute to the development of LC [97]. In other systems, MMP-9 has been linked to various conditions, including neuroinflammation and lung diseases [97,104,195]. It was also suggested that anti-idiotype antibodies directed against the ACE2 receptor might have been induced following SARS-CoV-2 infection and vaccination, potentially contributing to the neurological autoimmune manifestations of LC [196]. However, the potential pathogenic molecular mechanisms by which the persistent SARS-CoV-2 Spike protein, or the induced Spike protein-specific antibodies, would cause some pathophysiology of LC, whether following infection or vaccination, remain to be proven [157,158].

### 5.2. Residual Nucleoprotein and Other Viral Antigens in Patients with LC

A lingering SARS-CoV-2 nucleoprotein was detected using immunohistochemistry in the gastric and gallbladder tissues of patients with LC 274 to 380 days after acute infection resolved [107,108]. Using RNAscope, the presence of vRNA expressing nucleoprotein was detected in the appendix, skin, and breast tissues of two patients with LC, 163 and 426 days after the resolution of acute GI symptoms [105]. Using conventional immunohistochemistry, the nucleoprotein was also detected in the gut, colon, appendix, ileum, hemorrhoid, liver, gallbladder, and lymph nodes from five patients who recovered from COVID-19, up to 180 days after testing negative for SARS-CoV-2 [107,108,121]. Using multiplex immunohistochemistry, residual nucleoprotein was colocalized with CD68^+^ macrophages in the appendix, skin, and breast tissues of two patients with LC, 163 and 426 days after the resolution of acute symptoms [105]. An endoscopy study performed in 46 patients with inflammatory bowel disease (IBD), 219 days after a confirmed COVID-19 infection, revealed that the viral nucleoprotein persisted in the gut epithelium in 24 out of 46 patients despite the inability to culture SARS-CoV-2 from the gut tissue of patients with viral antigen persistence [107,108]. The study reported that LC symptoms in the majority of these IBD patients correlated with persistence of residual viral antigen, but not with those without viral antigen persistence. This study suggests that SARS-CoV-2 antigen persistence in infected tissues may be involved in LC symptoms, possibly by inducing T cell exhaustion, inflammation, and immune perturbation. Months after acute COVID-19 resolved, nasal cytobrushes, nasal washes, and tonsillar tissue fragments obtained from 48 children with LC undergoing tonsillectomy were tested using IHC, which revealed the presence of nucleoprotein on the epithelial surface and in lymphoid cells in both extrafollicular and follicular regions, as well as in adenoids and palatine tonsils. This suggests that tonsils and adenoids are significant sites of persistent viral protein in children [109]. Residual nucleoprotein and Spike proteins were detected in the plasma of children with MIS-C up to 62 days after resolution of acute COVID-19 [125]. Residual nucleoprotein was detected in patients with LC 12 to 16 months after COVID-19 resolved [117,128,167].

Immunofluorescence analyses of intestinal biopsies obtained from patients with LC 4 months after the onset of COVID-19 revealed the persistence of nucleoprotein in the small bowel of 7 out of 14 individuals, consistent with antigen persistence [124]. Nucleoprotein antigen was retained in gastric and gallbladder tissues of patients with LC for months to years after acute infection was resolved. Residual SARS-CoV-2 viral antigens are detected in the gastrointestinal, hepatic, and other tissues from patients with LC [105,107,108,121]. Months after the acute infection is resolved, nucleoprotein is detected in adenoid tonsils, adenoid tissue, nasal cytobrush, and nasal washes from children. It is important to note that most of the studies above are association and correlational studies that do not directly implicate residual Spike protein or Nucleoprotein with LC symptoms.

There exist imitations in identifying residual viral antigens in patients with LC, including their relatively low abundance and the lack of biomarkers for identifying infected cells that express viral antigens in vivo. The growing body of literature that demonstrates persistent virus and vRNA reservoirs within various body tissues, along with their correlation with LC symptoms, suggests a continuous production of SARS-CoV-2 antigens in patients with LC. Patients with LC exhibited persistent systemic inflammation 12 months after the acute COVID-19 episode, characterized by increased circulating levels of organ-damage markers, suggesting a persistent antiviral immune response [119]. For instance, elevated levels of organ damage markers, such as C3 protein and anti-nuclear autoantibodies, were detected in LC patients, indicating persistent immune activation associated with tissue or organ injury [119]. This suggests a sustained immune response, possibly driven by lingering virus or vRNA elements or immune dysregulation, even after initial recovery from acute infection [119]. However, it remains to be confirmed whether the various symptomatology and inflammatory signatures of LC are a direct consequence of specific antigens that are persistently and continuously stimulating the immune system. It is likely that the profile of antigens and epitopes targeted by T cells during LC differs from those targeted by T cells in acute COVID-19. The profile of antigens and epitopes targeted by tissue-resident T cells during LC may also vary depending on the affected organs.

Given the host specificity of SARS-CoV-2, few animal models accurately reproduce the natural course of viral infection, virus reservoirs, and the clinical symptomatology of LC [120,197,198,199,200,201,202].

## 6. Animal Models of LC to Study Persistent Reservoirs of Virus and Viral RNA (vRNA)

**Figure 5:**
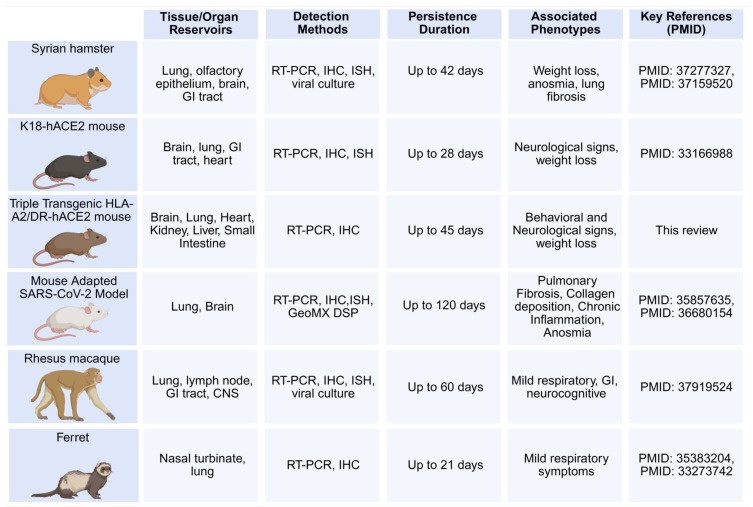
Animal models to study the role of the virus reservoir in Long COVID symptomatology and to test drug and immune therapeutics. Animal models of LC include mice [205,206,207]. ACE-2 transgenic (Tg) mouse models are widely used to determine the virus reservoirs [208]. HLA-A*02:01 and HLA-DR1 and ACE-2 triple transgenic mouse model susceptible to SARS-CoV-2 infections that develop ‘human-like T cell responses to HLA-restricted human CD4^+^ and CD8^+^ T cell epitopes and quantifiable virus and vRNA reservoirs in multiple organs [209,210]. The Golden Syrian hamster model exhibits natural susceptibility to SARS-CoV-2, making it an excellent model for investigating the pathophysiology of LC and long-term establishment of virus reservoirs in multiple organs [120]. The ferret model is ideal for studying virus reservoirs in the lungs as the respiratory tract structure closely resembles that of humans. Non-human primates (NHPs) may be used to understand the clinical manifestations of LC and to validate the safety and effectiveness of therapeutic candidates for clearing virus reservoirs and treating LC symptoms. This Figure is created using BioRender.

The importance of animal models as valuable and cost-effective tools for analyzing the virus reservoir and subsequently pre-clinical testing of drugs and immunotherapeutic candidates to eliminate the virus and vRNA reservoirs needs to be underscored [120,197,198,199,200,201,202] (Figure 5). We discuss the advantages and limitations of various animal models for studying viral persistence, with an eye to using these models to test antiviral and immune-based therapeutics [120,197,198,199,200,201,202]. Animal models may help address questions such as where, how, when, and under what circumstances persistent viruses and vRNA reservoirs can be detected and eliminated [120,197,198,199,200,201,202]. Animal models of LC can provide a means to quickly screen candidate treatments, yielding a strategy for rapid optimization and prioritization (as reviewed) [89,120,197,198,199,200,201,202]. Costly and lengthy clinical trials can require months to years to obtain definitive readouts on chronic inflammatory manifestations [46,203,204]. Because the biomarkers and diagnostic tests for LC disease phenotypes remain poorly defined, animal models that are robust and reliable may be crucial for evaluating therapeutic modalities and identifying the underlying cellular and molecular mechanisms and biomarkers of chronic LC [29,89,202].

Animal models are more likely to yield biomarkers for LC than humans because they allow controlled, longitudinal studies with consistent genetic backgrounds [139,211,212]. Unlike humans, animal models enable systematic analysis at serial time points post-infection, including histopathology in multiple organs, viral and vRNA persistence, and multi-omics assessments, to identify consistent pathological and molecular changes linked to LC symptoms [139,211,212]. Better-controlled comparisons between animals with and without LC, which reduce confounding factors such as genetic heterogeneity and comorbidities, are also present in human studies [139,211,212]. Animal models offer a tractable approach to identifying early, reproducible molecular signatures and pathological correlates that may later translate into human biomarkers [139,211,212]. Thus, while human biomarkers for LC remain elusive, animal models provide a powerful toolkit for identifying potential biomarker candidates under tightly controlled experimental settings [139,211,212].

**Figure 6:**
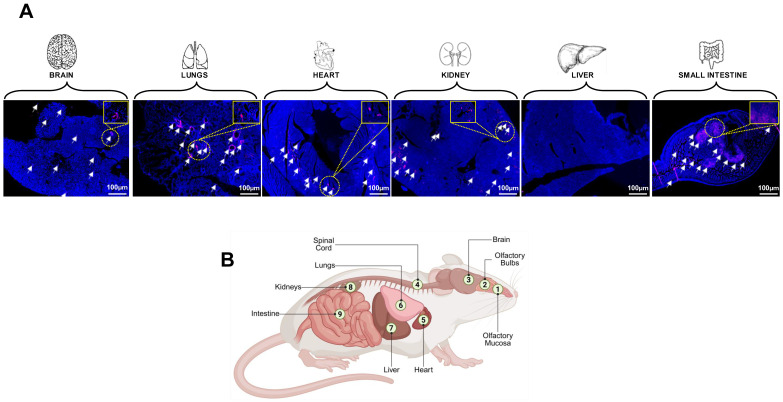
SARS-CoV-2 Reservoirs in multiple organs of ‘Humanized” HLA/ACE-2 triple Tg mice with Long COVID: (**A**) Immunohistochemistry (IHC) sections of the brain, lungs, heart, kidney, liver, and small intestine were collected on day 45 post-infection with 1 × 10^4^ PFU of the Delta variant (B.1.617.2) from the severe LC group of HLA/ACE-2 triple Tg mice and stained with SARS-CoV-2 Nucleoprotein protein antibody. White arrows indicate the expression of SARS-CoV-2 Nucleoprotein in these different organs of severe LC mice. Arrows point to the virus. Data shown at 4x magnification and 20x magnification (in inner box). Most persistent virus reservoirs are visualized in the brains, lungs, and guts of HLA/ACE-2 Tg mice with severe Long COVID. Persistent virus reservoirs and vRNA expressing the nucleoprotein in the lungs, brain, liver, heart, olfactory bulb, tonsils, kidney, and gut tissues of SARS-CoV-2 Delta variant-infected triple Tg mice can also be detected by qPCR [31,36,104] or quantified by digital droplet PCR (ddPCR) from formalin-fixed paraffin-embedded (FFPE) tissue block [31,36,126]. (**B**) Illustration of the organs with virus, vRNA, and viral antigen reservoirs in mouse models. Panel B is created using BioRender.

**Figure 7:**
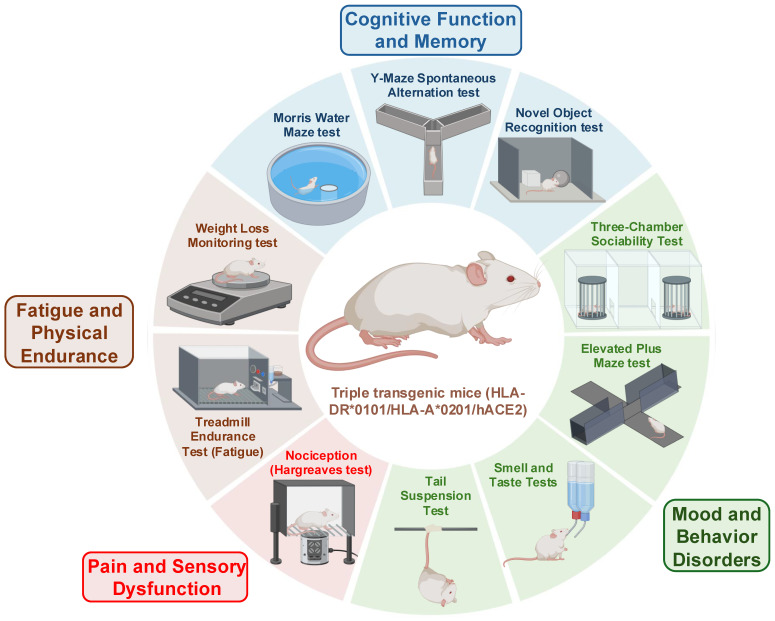
Illustration of ten experimental weight loss, behavioral, cognitive, and physical endurance tests to gauge the severity of LC in SARS-CoV-2-infected HLA/ACE-2 Tg mice. The behavioral tests include the following: (1) The Tail Suspension Test, which measures a state of “learned helplessness” or depression-like behavior similar to that observed in LC patients. (2) The Y-Maze Test (YMT) commonly used to assess memory impairment and specifically issues related to spatial working memory, cognitive flexibility, and exploratory behavior in mouse models. Spatial working memory is a cognitive function that enables individuals to hold and manipulate spatial information in their minds over time. (3) The Three Chamber Social Test, which measures social recognition and memory. (4) The Three Chamber Social Novelty Test, which measures social interaction, social memory, and social preference in mice [213,214,215,216,217]. This figure is created using BioRender.

Mice are the most widely used small animal model of LC due to their small size, short reproductive time, and the extensive list of related cell biology, genetic, and immunological tools, and reagents. Additionally, many LC disease symptoms, genetic susceptibility loci/genes, and phenotypic outcomes are highly reproducible between mice and humans [205,206,207]. The ACE-2 transgenic (Tg) mouse model, which possesses a well-characterized immune system, is widely used to determine the virus reservoirs and to study the role of specific genes and pathways thought to be involved in the pathophysiology of LC [208]. We recently generated a novel triple Tg mouse model of LC overexpressing human ACE-2 and human HLA class I and class II (i.e., HLA-A*0201 and HLA-DR1). This model has four attributes: (1) susceptibility to SARS-CoV-2 infections [209,210]; (2) development of both pulmonary, behavioral and neurological manifestations that mimic those seen in patients with LC; (3) development of T cell responses to HLA-restricted human CD4^+^ and CD8^+^ T cell epitopes from multiple SARS-CoV-2 antigens; (4) quantifiable virus and vRNA reservoirs in multiple organs. We detected persistent virus reservoirs and vRNA expressing the nucleoprotein in the lungs, brain, liver, heart, olfactory bulb, tonsils, kidney, and gut tissues of SARS-CoV-2 Delta variant-infected triple Tg mice 45 days post-infection by qPCR (Figure 6) [31,36,104]. Persistent vRNA can also be quantified by digital droplet PCR (ddPCR) from formalin-fixed paraffin-embedded (FFPE) tissue blocks [31,36,126], and (5) evaluation of the immunopathology, neuro-inflammation, and neuropathology linked to decreases in recognition memory, as measured using various established behavioral and cognitive tests, as illustrated in Figure 7 [213,214,215,216,217]. Thus, the triple Tg mouse model of LC enables us to test the protective efficacy of tissue-targeted drug and immunotherapy candidates, as well as to investigate the putative mechanisms driving the long-term respiratory, cognitive, and behavioral manifestations of LC. The Golden Syrian hamster model exhibits natural susceptibility to SARS-CoV-2, making it an excellent model for investigating the pathophysiology of LC and long-term establishment of virus reservoirs in multiple organs [120]. However, unlike mice, the use of hamster models is limited by the lack of immunological and genetic tools, which restrict the ability to perform advanced immunopathological studies [120]. The ferret model is ideal for studying virus reservoirs in the lungs as the respiratory tract structure closely resembles that of humans. Ferrets exhibit a natural susceptibility to SARS-CoV-2; however, the limited availability of ferret immunological and genetic tools also restricts the use of this model. Non-human primates (NHPs) are critical for understanding the clinical manifestations of LC and for validating the safety and effectiveness of therapeutic candidates for clearing virus reservoirs and treating LC symptoms. However, the use of NHPs is associated with high costs, limited availability, and complex handling requirements.

## 7. The Path Toward Therapeutics to Target and Clear the Virus and vRNA Reservoirs, and Cure LC

Solving the ‘SARS-CoV-2 reservoir problem’ may be key to achieving a cure (or at least a persistent remission) for many patients with LC. The molecular drivers and biomarkers of LC symptoms may be heterogeneous and remain poorly defined [218]. Thus, industry engagement in developing therapeutics has been limited [29,31,33,34,35,36,117]. Unfortunately, the current landscape of clinical trials for LC is primarily observational, designed to understand the pathophysiology of LC [23]. There are a limited number of clinical trials testing drug therapies, and a notable lack of T cell-based immunotherapies.

Treating the persistent virus reservoir in LC may be informed by previous clinical trials targeting persistent virus reservoirs in other RNA viruses, such as human immunodeficiency virus (HIV) and hepatitis C. The viral reservoir in HIV and HCV is well-established and characterized by the persistence of replication-competent virus in specific cellular and tissue compartments [219,220]. In HIV, the reservoir consists primarily of latently infected CD4^+^ T cells and other immune cells, with integrated proviral DNA that can reactivate and produce infectious virus. This virus reservoir is stable and quantifiable, and its dynamics have been extensively studied [221]. In contrast, the dynamics of the viral reservoir in LC remain to be identified, and the clinical significance of persistent SARS-CoV-2 in immunocompetent individuals should be further characterized [222]. Moreover, while vRNA has been widely reported, the presence of replication-competent virus has not been consistently demonstrated, and the mechanisms of persistence remain unclear [222]. There is an association between viral RNA persistence and LC symptoms, but causality and the role of these reservoirs in pathogenesis remain to be established [222]. Key gaps include the development of standardized biomarkers for reservoir detection and the assessment of the impact of antiviral and immunotherapeutic interventions on reservoir clearance and symptom resolution.

### 7.1. Antiviral Therapies for LC

Antiviral agents like remdesivir, molnupiravir, and nirmatrelvir–ritonavir (Paxlovid) have been tested in LC with limited efficacy [23,48,52,223]. Three clinical trials have shown that administering the antiviral drug Paxlovid during COVID-19 infection yields a modest benefit in reducing the likelihood of LC [31,203,224]. However, a recent study with a large, nationally sampled cohort, a contemporary study period, and causal inference methodology, found that Paxlovid treatment during acute COVID-19 had no effect on subsequent LC incidence [225]. Some improvement in LC symptoms was reported following tocilizumab administration, especially in cases with elevated inflammatory markers [226]. JAK inhibitors have also shown promise, with recent clinical trials indicating their effectiveness in modulating the immune response and reducing the severity of persistent symptoms [227]. Molnupiravir has also been tested for lowering symptom duration and severity in patients with LC [228,229]. Early initiation of drug treatment during SARS-CoV-2 acute infection reduces virus load and is likely beneficial for LC patients, as it may have contributed to lowering virus reservoirs [230,231,232,233,234].

### 7.2. Immune Therapies to Eliminate or Reduce Persistent Virus and vRNA Reservoirs in LC

In the absence of antiviral drug therapy, virus-specific CD4^+^ and CD8^+^ T cells play a central role in controlling and suppressing viremia, virus reservoirs, and viral RNA reservoirs. While T cells appear vital in clearing the virus reservoir, to the best of our knowledge, there are currently no clinical trials for T cell-based immunotherapy that would function to clear or reduce virus reservoirs, thereby reversing the inflammatory, cognitive, and behavioral symptoms of LC. A tissue-targeted T cell-based immunotherapy that boosts functional tissue-resident T_RM_ cells could reduce persistent virus reservoirs, thereby addressing both systemic and organ-specific manifestations of LC.

Besides inducing or boosting the frequency and function of T cells, immunomodulatory treatments have also gained traction as a cornerstone of LC management. Cytokines, Including Interferon-Gamma (IFN-gamma), IFN-gamma-induced protein 10 (IP-10), tumor necrosis factor (TNF), IL-1beta, IL-2, IL-4, IL-6, IL-8, IL-10, IL-12, and IL-17A, induced by SARS-CoV-2 infection, play a crucial role in the pathophysiology and progression to LC [189]. Treatment of LC with a SARS-CoV-2 antiviral and IL-6 blockade in a patient with rheumatoid arthritis and SARS-CoV-2 antigen persistence has been reported [106,235,236,237,238,239,240]. The study demonstrates transient disappearance of antigen persistence and decreased antiviral and autoimmune T cell responses after nirmatrelvir/ritonavir and tocilizumab treatment. The seed meal of *Perilla frutescens* (*P. frutescens*), which contains two primary dietary polyphenols (rosmarinic acid and luteolin), has been suggested to modulate Spike protein S1 subunit-induced lung inflammation during LC [104,194,241,242,243,244,245]. Immune dysregulation associated with persistence of subunit S1 of the Spike protein was detected in patients with LC 8 months after COVID-19 resolved [167]. Whether the host tissue-resident CD4^+^ and CD8^+^ T cells affect the size, clonality, cellular, tissue, and organ distribution of the virus reservoir and viral RNA reservoirs remains to be determined.

The hypothesis of persistent Spike protein in patients with LC following vaccination and/or infection, together with the lack of current Spike protein-based COVID-19 vaccines to induce long-lasting protection against disease, ongoing viral transmission, or future CoV outbreaks, has raised questions about whether future immunotherapies to clear the virus reservoirs in LC should or should not include the Spike protein. Spike protein mRNA vaccines appeared to induce IgG4, whereas Spike protein vaccines and SARS-CoV-2 infection both promote IgG2. Emerging evidence suggests that an increase in IgG4 levels detected after mRNA vaccines may constitute an immune tolerance mechanism to the Spike protein, which could encourage SARS-CoV-2 infection and replication by suppressing natural antiviral responses [246]. Spike protein-induced cross-reactive IgG4 antibodies may contribute to autoimmunity, another pathophysiological mechanism that may lead to LC [247]. Suppose the Spike protein needs to be included in future immunotherapies to clear the virus reservoirs in LC. In that case, it is recommended to use the S1 subunit, which does not elicit IgG4 antibody responses, while still inducing T cell responses that contribute to clearing the virus reservoir.

Because Spike also induced both CD4^+^ and CD8^+^ T cell responses, and since most individuals (70%) have already received at least one dose of Spike protein-based COVID-19 vaccines, it is likely that Spike-specific memory CD4^+^ and CD8^+^ T cells have been developed and often boosted and re-boosted following multiple exposures to various SARS-CoV-2 variants over time [248,249]. Since some Spike protein-vaccinated patients still develop LC, some Spike protein-specific memory CD4^+^ and CD8^+^ T cells may be pathogenic by producing excessive cytokines, rather than protective, as observed in other systems [250,251,252,253,254,255,256,257]. For instance, a subset of Spike protein-specific pathogenic Th_1_ and Th_17_ cells can produce large amounts of inflammatory cytokines, such as IL-1 or IL-17, which can damage tissues and worsen inflammation in LC, thus contributing to the harmful effects of SARS-CoV-2 infection, rather than helping to clear it, as shown in other systems [258,259,260,261,262,263,264,265]. Cytokines, Interferon-gamma (IFN-gamma), IFN-gamma-induced protein 10 (IP-10), tumor necrosis factor (TNF), IL-1beta, IL-2, IL-4, IL-6, IL-8, IL-10, IL-12, and IL-17A, induced by SARS-CoV-2 infection, play a crucial role in the pathophysiology and progression to LC-19 [189]. In severe cases of COVID-19, specific T cell subsets, like pathogenic Th_1_ cells, can contribute to lung damage and inflammation [104,251]. Thus, while T cells are crucial for fighting off SARS-CoV-2 infections and clearing virus reservoirs [248,249], it is also possible that subsets of Spike protein-specific T cells can be detrimental, leading to tissue damage or exacerbating LC symptoms [258,266,267,268,269,270,271,272,273,274,275,276,277,278,279,280,281,282,283,284,285,286,287]. Moreover, patients with LC who harbor a persistent virus or vRNA reservoir may continuously produce the Spike protein and other antigens, overstimulating Spike protein-specific T cells that become exhausted and lose their ability to effectively fight the virus and clear the reservoirs. In many chronic and persistent viral infections like HIV or hepatitis, T cells can become exhausted, leading to persistent infection and disease progression. Based on the above assessment of Spike protein B- and T cell response following infection and vaccination, it was suggested not to include Spike protein in any immunotherapy to clear the virus reservoir in LC.

With no approved treatments, the long-term global health and economic impact of chronic LC remains high and growing [23,30,31,288]. Despite these advances, challenges persist in the development and implementation of T cell therapies for LC, including the heterogeneity of symptoms, the absence of standardized diagnostic tests, and the necessity for prolonged clinical trials to assess the efficacy and safety of T cell treatments [29]. Due to a lack of a specific biomarker for LC, industry engagement in developing therapeutics has been limited [29,31,33,34,35,36,117].

## 8. Conclusions

- A potential causative factor of LC, in a large subset of patients, is that reservoirs of virus and/or viral RNA (vRNA) or fragments may persist and replicate in multiple sites of the body, which may drive chronic inflammation and provide continuous viral antigenic stimuli to exhausted CD4^+^ and CD8^+^ T cells [31,33,34,35,36]. However, other hypotheses regarding the causative factors of LC include metabolic disturbances, immune dysbiosis, micro-clotting, autonomic dysfunction [38,43,45,46,47], and the reactivation of other non-SARS-CoV-2 viruses, such as HSV-1, HSV-2, EBV, CMV, and HHV-6, which may be a driver of LC [48,49].
- While a growing body of literature has shown that persistent virus and vRNA reservoirs within cells from various body tissues correlate with some of the LC symptoms, it remains to be confirmed whether the various symptomatology of LC and pro-inflammatory signatures are a direct consequence of persistent viral antigens.
- Although viral persistence may be linked to inflammation and immunological overactivation in patients with LC, the underlying mechanism of such stimulation remains to be fully elucidated. Nevertheless, SARS-CoV-2-derived vRNA and protein antigens (i.e., Spike protein and Nucleoprotein) appeared to be released in various organs (e.g., gut, brain, heart, and reproductive organs) and in the circulation, possibly inducing inflammation and T cell exhaustion that persists months after the acute COVID-19 infection [23,35,38,39,40,41,42]. This suggests at least one immune evasion mechanism by which the virus may establish its reservoir in LC patients.

## 9. Future Directions

- Knowledge about chronic LC and its lingering health effects, months and years following acute infection, is still in its embryonic stage. Currently, there are more questions than answers regarding the underlying mechanisms by which the virus and vRNA persistence may lead to the symptomology of LC, as well as how to reverse this outcome.
- Future research should aim to develop reliable animal models that more accurately replicate virus reservoirs and the symptoms of LC in humans. As with most diseases, no single animal model can fully replicate LC as it occurs in humans; however, studies conducted on different species may yield biomarkers and help develop drugs and immunotherapies for LC.
- The integration of multi-omics approaches, including genomics, proteomics, and metabolomics, can provide a more comprehensive understanding of symptomologies of LC. Enhanced efforts to model chronic symptoms, combined with the implementation of artificial intelligence, deep learning, organoids, and organ-on-chip models, will further advance the field, enabling more precise and effective therapeutic strategies for LC.
- While growing evidence suggests that persistent virus and viral vRNA detected in patients with LC may produce consistent antigenic stimulation [23,35,38,39,40,41,42], it remains to be determined whether persistent virus and vRNA reservoirs consistently express residual viral antigens in multiple organs and circulation (e.g., Spike protein and Nucleoprotein), and whether this is directly responsible for the chronic inflammation, as well as T cell dysfunction/exhaustion associated with LC symptoms. This will require large LC patient and control groups, as well as reliable animal models of persistent virus and vRNA reservoirs associated with LC-like symptoms, as seen in humans [120].
- The mechanism by which residual Spike protein, S1 subunit, and other SARS-CoV-2 antigens may persist in the plasma and other organs of some patients remains to be explored. While persistent Spike protein has been detected in some patients with LC, the finding should be regarded for now as an association, rather than a cause-and-effect relationship [117]. Whether Spike or any residual SARS-CoV-2 antigen contributes to chronic inflammation and T cell exhaustion that led to LC symptoms requires investigation in large LC patient and control groups, as well as in reliable animal models of LC using multiple pathophysiological and neuro-immunological approaches [120].
- There remains an urgent need to develop drugs or immunotherapeutic strategies that clear persistent virus and vRNA reservoirs. This will likely contribute to curbing the symptoms that target twelve major organ systems, causing dyspnea, vascular damage, cognitive impairments (“brain fog”), physical and mental fatigue, anxiety, and depression in at least a subset of patients with LC. This significant gap in our knowledge will likely require the development of a tissue-targeted immunotherapeutic strategy that increases the frequency and function of antiviral CD4^+^ and CD8^+^ TRM cells within affected tissues, thereby clearing persistent virus reservoirs and alleviating symptoms of LC.
- We are currently investigating the mechanisms by which SARS-CoV-2 causes immune dysfunction and contributes to the progression of LC disease. Information gained from these studies will be crucial to the development of novel immune therapies for treating LC. In a ‘humanized” mouse model of LC, we are examining the PD-1, TIM-3, PSGL-1, and/or LAG-3 blockade approach as a potential target for purging the virus reservoirs (Figure 5, Figure 6 and Figure 7). One goal is to utilize this knowledge to design strategies for enhancing the efficacy of immune therapy in patients with LC.
- Our ultimate and long-term goal is to identify protective T cell antigens and epitopes that are preferentially recognized by CD4^+^ and CD8^+^ T cells from patients who have resolved acute COVID-19 and never developed LC (recovered asymptomatic patients). These protective T cell antigens and epitopes will then be used to design a T cell immunotherapeutic strategy, such as the recently described Prime/Pull/Keep immunotherapy recently developed for other viral pathogens [289,290], to boost strong and long-lasting tissue-resident SARS-CoV-2-specific CD4^+^ and CD8^+^ TRM cells, that will then clear or reduce the persistent virus and vRNA reservoirs, and reverse chronic inflammatory and severe symptoms of LC.
- To treat LC patients with T cell immunotherapy, one would first need to select the subset of LC patients who exhibit persistent virus and vRNA reservoirs detected, either directly using ultrasensitive assays to trace virus, or vRNA, or residual viral proteins from, blood, stool, and gut/rectum biopsies or indirectly through virus-specific B and T cell responses, in patients with LC [105,119,125,291,292,293,294,295]. SARS-CoV-2 protein fragments (such as Spike, nucleoprotein, and other viral proteins) are found in the blood of many patients with LC using highly sensitive tests like Simoa (Single Molecule Array) [117,292,293]. Virus vRNA and proteins can also be detected in biopsies of the gut, rectum, tonsils, and tongue [105,111,119,125,291,292,294,295]. Biomarker-guided trials have emerged as a cornerstone of future research efforts and may be a promising approach for personalized medicine in LC [218]. In the future, a combination of biomarkers—blood-borne viral proteins and persistent viral vRNA in stool—is being investigated as a potential diagnostic test to identify LC patients with viral reservoirs [117,296,297]. However, many of these methods are still under clinical development, and no single test has been universally confirmed. Nevertheless, early results are promising for differentiating patients with LC who have underlying viral persistence from those with other causes.
- Treating LC presents a unique set of challenges, including the heterogeneity of symptoms and lack of specific biomarkers and diagnostic tests [29,218]. This variability not only complicates patient selection but also makes it difficult to establish uniform treatment protocols [218]. This heterogeneity may necessitate a more nuanced approach to trial design, incorporating stratified analyses and subgroup-specific interventions to address the diverse patients with LC.
- Since LC is present in various pathophysiology and clinical presentations, patients with LC may respond differently to treatment. While a large subset of patients with LC appear to express persistent reservoirs of virus, vRNA, and/or residual viral proteins, the general utility of T cell-based immunotherapy relies on the proportion of LC patients for whom these reservoirs are the etiology of the disease. However, a T cell immunotherapy that targets T cell antigens selected as being preferentially recognized by the immune system in patients who recovered by clearing acute infections and never progressed to LC (i.e., recovered, or “asymptomatic” patients) may prevent progression to LC. Hence, this strategy may also be effective as a post-exposure prophylaxis treatment for preventing LC.
